# Dysphagia caused by focal guttural pouch mycosis: mononeuropathy of the pharyngeal ramus of the vagal nerve in a 20-year-old pony mare

**DOI:** 10.1186/2046-0481-66-13

**Published:** 2013-07-11

**Authors:** Annemarie Eichentopf, Alice Snyder, Stephan Recknagel, Albrecht Uhlig, Veronika Waltl, Gerald Fritz Schusser

**Affiliations:** 1 Department of Large Animal Medicine, Faculty of Veterinary Medicine, University of Leipzig, An den Tierkliniken 11, 04103 Leipzig, Germany; 2 Institute of Pathology, Faculty of Veterinary Medicine, University of Leipzig, An den Tierkliniken 33, 04103 Leipzig, Germany

**Keywords:** Dysphagia, Guttural pouch mycosis, Regurgitation, Vagus nerve, Pharyngeal branch

## Abstract

A 20-year-old pony mare was presented to the equine hospital with a ten-day history of dysphagia, regurgitation and coughing. An obstruction of the oesophagus was excluded via endoscopy, but the proximal oesophagus appeared to be distended and circular contractions were missing. A guttural pouch endoscopy revealed a single, black-mottled plaque on the pharyngeal ramus of the vagus nerve in the left guttural pouch, causing a local swelling of this nerve. The pharyngeal ramus seemed to be atrophic distal to the lesion. A biopsy was taken from the lesion and histopathological findings proved the reasonable suspicion of a guttural pouch mycosis with a high degree of purulent-necrotic inflammation and invasion of fungal hyphae. There were no signs of neoplasia, such as melanoma. Daily guttural pouch irrigations with a clotrimazole emulsion (20 g Canesten^®^ Gyn^4^ solved in 500 ml water), led to a good recovery of the mucosa above the nerve. Periodic endoscopic examination of the left guttural pouch showed that local thickening and distal atrophy of this pharyngeal ramus did not improve, neither did the clinical symptoms. Due to progressive weight loss, acute respiratory distress and aspiration pneumonia, the 20-year-old pony mare unfortunately had to be euthanized three weeks after discharge. This case report emphasizes the enormous importance of a single nerve for the realization of the swallowing process. The one-sided loss of function of the pharyngeal branch of the vagal nerve cannot be compensated neither by the remaining ipsilateral nerves nor by the contralateral normal functioning glossopharyngeal and vagal nerves and thus inevitably leads to severe dysphagia.

## Background

The physiological swallowing process is realized by a complex sequence of over 20 head and neck muscles, involving numerous cranial nerves (V, VII, IX, X, XI, XII). The glossopharyngeal (IX), hypoglossal (XII) and vagal nerve (X) are the most important for the innervation of the pharynx [1,2]. A loss of function of these nerves will cause neurologic dysphagia. Focusing on this case, dysphagia combined with feed-containing nasal discharge from both nostrils is usually a sign of serious pharyngeal, laryngeal or esophageal disease [1]. Traumatic, mechanical, congenital, inflammatory, infectious, and non-infectious reasons that could cause dysphagia need to be excluded [1,3,4].

The pharyngeal branch of the vagus nerve is the most important for the motor innervation of the pharyngeal muscles [2,5]. This sensomotor nerve innervates the palatinus, levator veli palatini, and palatopharyngeus muscles, as well as the dorsal pharyngeal constricting muscles, which include the cricopharyngeus, thryopharyngeus, pterygopharyngeus, and hyopharyngeus muscles. Those constricting muscles are essential for the swallowing process because they are needed to propel feed into the oesophagus [6]. The pharyngeal branch of the vagus is also responsible for sensory innervation of the pharynx and larynx and is, thus, essential to activate the swallowing reflex [6]. In contrast, the glossopharyngeal nerve provides sensory innervation to the nasopharynx, specifically to the dorsal and lateral walls of the pharynx, as well as to the nasopharyngeal surface of the proximal half of the soft palate, and supplies motor innervation only to the stylopharyngeus muscle, a muscle which contracts during swallowing and dilates the pharynx [7].

From an anatomic point of view, the glossopharyngeal and vagal nerves act closely together. These two nerves, including the accessory nerve (XI), belong to the so-called “vagal group” [2]. They emerge from the same area of the medulla oblongata, exit the skull through the foramen lacerum and run along the outside of the guttural pouch enfolded in its thin mucous membrane [1,2,5]. Because these nerves shine through the mucosa of guttural pouch, they can be seen and evaluated endoscopically. The vagal nerve, accompanied by the hypoglossal nerve (XII) and the internal carotid artery, runs through a fold of mucous membrane, which is described as the plica neurovasculosa. This mucous membrane fold is an excellent landmark for endoscopic examination, because it divides the medial compartment of the guttural pouch into a medial and lateral recess [1,8]. The glossopharyngeal nerve leaves the plica neurovasculosa approximately half-way through its total length, to continue coursing in a lateral direction along the stylohyoid bone, whereas the hypoglossal nerve (XII) continues in a rostral direction to innervate the tongue [2]. The pharyngeal branch of the vagal nerve exits the plica neurovasculosa medioventrally to enter into the pharyngeal muscles [1]. Together with the pharyngeal branch of the glossopharyngeal nerve, it will finally create the pharyngeal plexus [1,2,5]. This case report presents a one-sided neuritis of the pharyngeal branch of the vagal nerve caused by a small fungal plaque.

## Case presentation

### Case history

A 20-year-old grey pony mare was presented to the Department of Large Animal Medicine of the University of Leipzig in the late spring of 2012 with a ten-day history of dysphagia, regurgitation and coughing. The 390 kg German Riding Pony also showed fever (up to 40.0°C) and had already been treated with antibiotics and anti-inflammatory drugs by the referring veterinarian. The mare was kept on pasture together with another pony and was used for carriage riding. The owners had not noted any abnormalities or impaired performance before those ten days.

### Clinical findings

The physical examination confirmed that the horse was bright, alert, responsive, and in good condition, even though it had a rectal temperature of 39.4°C. Heart and respiratory rate were within normal limits. Auscultation of the heart and abdomen revealed no clinical abnormalities. Wheezes were auscultated in both sides of the lung. The mucous membranes were pink and capillary refill time was 2 sec. The mandibular lymph nodes were enlarged, moveable and lobed, but not painful. Palpation of the parotid area and larynx was unremarkable. The coughing reflex occurred spontaneously and on provocation. At first, the pony showed serous nasal discharge from both nostrils without any food components. She also presented frequent spontaneous snorting, puffing and coughing. Later, after feeding, the pony showed severe dysphagia and increased nasal discharge containing lots of masticated food. Intense observation revealed that the mare masticated the food for a long time, which collected in her mouth forming cheek pouches. Then, the mare finally tried to swallow by stretching her neck and contracting the omohyoid and sternocephalic muscles. This was usually followed by an expiratory cough. Neurological examination of the cranial nerves, as well as inspection and palpation of the oral cavity and tongue, revealed no abnormalities. Passing a nasogastric tube was possible without any resistance.

Laboratory blood-testing showed an increased lymphocyte-neutrophil ratio 1:4.7 (reference range: 1:2), erythrocyte sedimentation rate 65/137 mm (reference range: <50/<100 mm), and GGT activity 114 U/l (reference range: 11–44 U/l), as well as a hyperfibrinogenaemia 6.8 g/l (reference range: 2–4 g/l).

**Figure 1:**
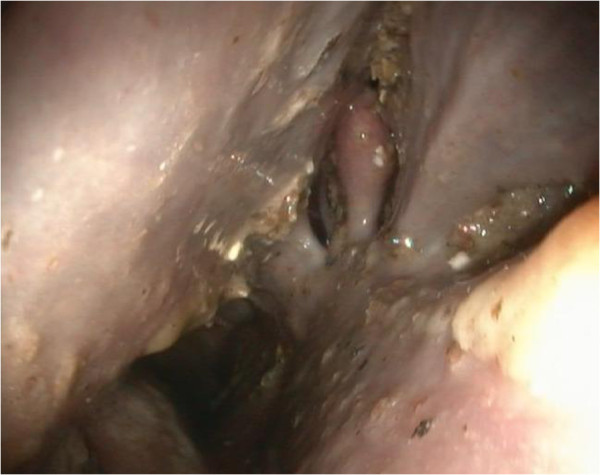
**Endoscopic view from the left ventral nasal passage to the left ethmoid**: **Masticated regurgitated feed in the ethmoid region and ventral nasal passage.**

**Figure 2:**
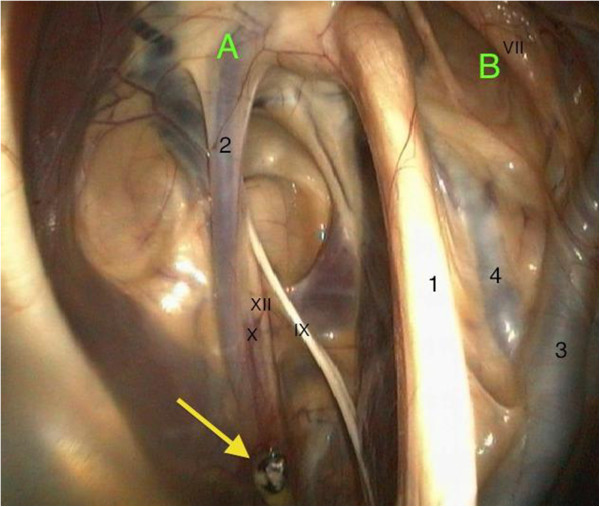
**Endoscopic view into the left guttural pouch: the medial compartment (A) is separated from the lateral compartment (B) by the stylohyoid bone (1).**  The plica neurovasculosa contains the internal carotid artery (2), the glossopharyngeal nerve (IX), the hypoglossal nerve (XII), and the pharyngeal branch of the vagus nerve (X) with the mycotic plaque (yellow arrow). The external carotid artery (3), the auricularis caudalis artery (4) and the facial nerve (VII) are prominent structures in the lateral compartment.

**Figure 3:**
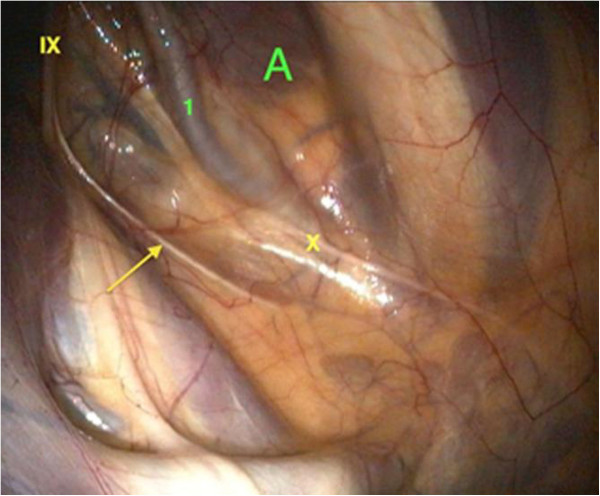
Endoscopic view into the right guttural pouch: the course of the pharyngeal branch of the vagus nerve (X), as well as pharyngeal branch (arrow) of the glossopharyngeal nerve (IX) and the internal carotid artery (1) within the medial compartment (A) of the right guttural pouch.

A thoracic radiograph of the pony was not taken due to money restrictions. Instead, an upper airway and guttural pouch endoscopy was performed subsequent to the physical examination. Therefore the pony was sedated with detomidine^1^ (0.02 mg/kg bw IV). The upper airway endoscopy revealed the following findings. The nasal passages were filled with masticated food (Figure 1), and the ethmoid bones and guttural pouch openings were normal. The larynx was symmetrical and the epiglottis seemed to be normal. The left ventral wall of the pharynx, directly in front of the epiglottis, appeared to be atonic, thus an accumulation of mucus, mixed with food particles, was detected. Parts of this fluid were able to float into the trachea without provoking a swallowing or coughing reflex. An instillation of water into the nasopharyngeal cavity did not lead to a physiological swallowing reflex. The proximal third of the oesophagus was distended and no circular constrictions were seen. The mucosa seemed to be normal. The other two thirds, including the cardia, had a normal circular muscle constriction and mucosa. The upper third of the trachea contained a small amount of saliva mixed with masticated food and bronchotracheal mucus. Endoscopy of the left guttural pouch revealed a small single black-mottled plaque on the pharyngeal branch of the vagus, causing it to swell (Figure 2). This plaque was completely removed via biopsy forceps for pathohistological examination. The pharyngeal ramus medioventral to the lesion seemed to be atrophic. The right guttural pouch was without pathological findings (Figure 3). All diagnostic investigations were consistent with optimal clinical case management and as such were not subject to a requirement for ethical approval.

### Histopathological findings

**Figure 4:**
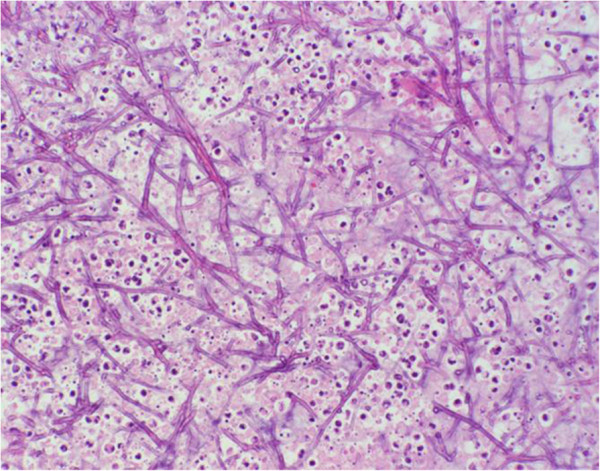
**Microscopic image of biopsy findings: biopsy with intralesional myriads of branched fungal hyphae.** H&E stain, x 20.

**Figure 5:**
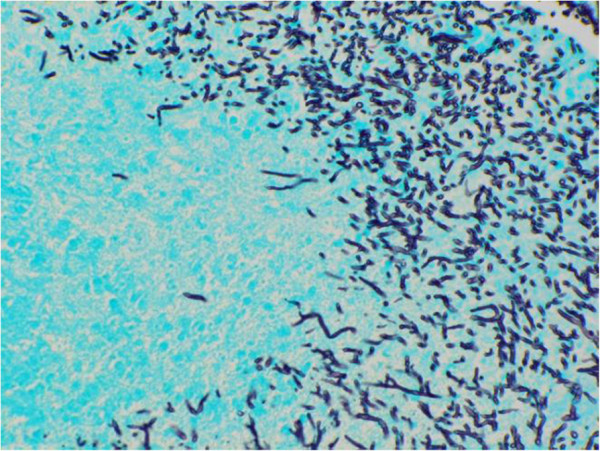
**Microscopic image of biopsy findings: biopsy with intralesional myriads of branched fungal hyphae.** Grocott’s stain, x 20.

The samples were fixed in 4% buffered formalin, embedded in paraplast, sectioned at 2–3 μm, and stained with haemalum and eosin (H.-E.) (Figure 4). Histopathological examination of the biopsy revealed severe chronic suppurative and necrotizing inflammation with intralesional evidence of myriads of dichotomous branched fungal hyphae. The fungal elements could be confirmed by the use of Grocott’s stain (Figure 5). The fungal organisms were recognized as Aspergillus species.

### Medical treatment

The pony was treated with antibiotics (Amoxicillin^b^, 10 mg/kg bw IV BID) and a non-steroidal anti-inflammatory drug (Metamizol^c^, 20 mg/kg bw IV BID) because of increased body temperature. The plaque was completely removed via guttural pouch endoscopy and the pony was sent home. After 14 days, when histopathological results were obtained, the pony was presented again. Unfortunately, the mare showed clinical deterioration, such as weight loss (12 kg), fever (39.7°C), aggravated breathing noise, and a mild leukocytosis (12.3 G/l; reference range: 5,4-10,0 G/l). Although the owners were informed about the poor prognosis, they insisted on the mare being treated therapeutically.

Therefore, we performed daily guttural pouch irrigations with a 0.08% clotrimazole^d^ emulsion (20 g solved in 500 ml water) over a time period of 14 days. The mare was sedated (detomidine^a^ 0.02 mg/kg bw IV) every morning and a guttural pouch catheter was placed via the nasal passages into the left guttural pouch. Before entering the guttural pouch, nasal passages were flushed with warm water. Subsequently the clotrimazole emulsion was instilled into the left guttural pouch and the catheter was removed.

Antibiotics were continued and a non-steroidal anti-inflammatory drug (Flunixin meglumine^e^, 1.1 mg/kg bw IV BID) was given. Under this medication, the pony’s appetite improved and body condition stabilized. The severity of the dysphagia, nasal discharge, coughing, and snorting remained very variable, but did not improve overall.

**Figure 6:**
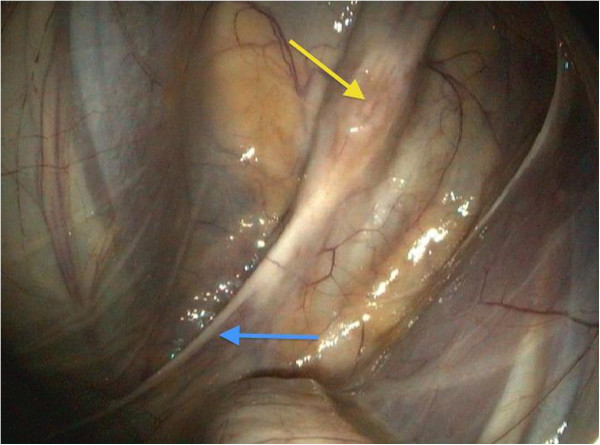
**Lesion after 14 days of daily irrigation with a 0.08 % clotrimazole emulsion: the mucous membrane above the lesion of the pharyngeal branch of the vagus nerve is completely healed (yellow arrow).**  The nerve itself remains thickened at the former site of the lesion and atrophic in distal parts (blue arrow).

Endoscopy of the upper airways and guttural pouches was repeated. The nasal passages were full of masticated food, the larynx was symmetrical, and the guttural pouch openings and epiglottis were normal. The left ventral wall of the pharynx still appeared to be atonic. Masticated food was seen in the upper third of the trachea. The proximal oesophagus was unchanged. The pharyngeal branch of the vagus in the left guttural pouch no longer showed any black-mottled plaques, thus the fungal lesion did not reappear. The mucous membrane of the guttural pouch covering the pharyngeal branch of the vagus was fully healed. There were no signs of inflammation and the granulation tissue had completely disappeared (Figure 6). Nevertheless, the nerve itself remained thickened proximally and atrophic distally.

Despite good care and excellent appetite the pony lost 31 kg during her stay at the hospital. When the mare was discharged, the owners were informed of the poor prognosis due to almost no improvement in clinical signs.

Unfortunately, the referring veterinarian had to euthanize the mare three weeks after discharge, due to acute respiratory distress, pneumonia, weakness, and further weight loss. Unfortunately, the pony was not sent to necropsy.

## Discussion

This case report presents an uncomplicated diagnosis of a rather minimal guttural pouch mycosis with a large impact on the quality of life and a life-threatening illness of a 20-year-old pony.

Guttural pouch mycosis is a rare, globally widespread, fungal disease with no predispositions to age, gender, breed, or regional origin [9]. However, it does appear more often in stabled than in pastured horses, especially during the warm months of the year [10]. This observation does not apply to the case presented and a New Zealand case report of six horses with guttural pouch mycosis [11]. Causal connections between the housing and feeding of affected horses need to be investigated more detailed in the future.

Pathogenesis of guttural pouch mycosis is still unknown. In most cases, Aspergillus spp., such as Aspergillus nidulans, Aspergillus fumigatus or Aspergillus flavus, are incriminated. Candida spp. can rarely be found [9,10,12,13]. Aspergillus spp. were considered to be the most probable cause of guttural pouch mycosis in this case because of the black-mottled appearance of the plaque and histopathological findings. Unfortunately, the specimen was not cultured, due to the small size of the plaque. Opportunistic fungi such as Aspergillus spp. can be naturally found in the environment and the upper airway tract of horses. Bad general condition, immunodeficiency, inflammation, or defects of the mucosal barrier can be initiating factors for fungal hyphae to attach to exposed mucosal fibrinogen [10,12,14,15]. Aspergillus spp. are considered to be angiotrophic, thus fungal hyphae often invade the internal carotid artery, normally causing life-threatening epistaxis [16]. An anatomical vicinity to the internal carotid artery was also noticeable in this case.

The second most common clinical sign of guttural pouch mycosis is dysphagia. The pharyngeal plexus, which contains nerve fibers of the pharyngeal branches of the glossopharyngeal and vagal nerve, is extremely important for the motor innervation of the pharynx. In a study released in 2005, it was shown that bilateral anesthesia of the glossopharyngeal nerves surprisingly did not lead to dysphagia [17]. Similar observations were made in dogs [18]. Thus, the pharyngeal branch of the vagus nerve is of major importance for the motor innervation of the pharynx [19]. This can be verified by this case report because only this nerve was affected.

The atonic ventral wall of the pharynx, with accumulated saliva in front of the epiglottis, is a sign of dysfunction of the levator veli palatini muscle, which elevates the soft palate and the palatinus and palatopharyngeus muscles, which move the caudal part of the soft palate during swallowing [6]. The pharyngeal branch of the vagus innervates all of these muscles. Another indication of vagal nerve involvement was reduced dilatation and motility of the upper part of the oesophagus, as oesophageal branches from this pharyngeal branch supply the muscularis of this region of the oesophagus [5].

Despite the partial pharyngeal paralysis, the pony was still able to communicate with other horses by vocalization. The mare did not show a laryngeal hemiplegia, which also proves a precise involvement of the pharyngeal branch of the vagus because laryngeal muscles are innervated by recurrent laryngeal and cranial laryngeal branches of this nerve [2,20]. This also explains the absence of laryngeal stridor.

Holcombe et al. (1990) were able to produce a reversible dorsal displacement of the soft palate by anesthetizing both pharyngeal branches (left and right) of the vagal nerve at the guttural pouch site [6]. However, the 20-year-old pony did not show a dorsal displacement of the soft palate. An involvement of the hypoglossal nerve is very likely because of its vicinity to the lesion. Although symptoms such as reduced tonicity of the tongue muscle, abnormal tongue movements or mastication and injuries on the tongue were not found in this case [1,19,21]. The neurological deficits were possibly compensated by the healthy side and, therefore, were not noticed.

Complications of acute respiratory distress and aspiration pneumonia were conceivable. An indication for a thoracic radiograph was given, but due to money restrictions we refrained from this. Obstruction of nasal airways by feed and the fact that horses are obligate nasal breathers contributed to these complications [6]. The evaluation of a thoracic radiograph would have emphasized the clinical findings, but would not have influenced the therapy and prognosis effectively [10].

The treatment of guttural pouch mycosis can be attempted by using topical and/or systemic antifungal medication [9]. According to Edwards and Greet (2007), a local treatment is indicated for neurological deficits [10]. Various antimycotics, such as nystatin, ketoconazole, miconazole, natamycin, enilconazole, clotrimazole in different pharmaceutical forms (powder, solutions), and thiobendazole, or irritant reducing solutions, such as povidone-iodine or 6% neomycin mixed with 1% gentian violet, can be used [9,10,12,14,22,23]. Unfortunately, a standard approach to the treatment of guttural pouch mycosis does not exist. In this case, we chose a topical therapy with a 0.08% clotrimazole emulsion, which led to an excellent recovery of the mucosa lying over the nerve with no signs of inflammation. The instillation of this emulsion had two advantages. Firstly Canesten^®^ Gyn^d^ is specifically made for mucosa-associated fungal infections and secondly due to its consistency it sticks to the wall of the guttural pouch leading to a more effective, long-lasting therapy. Nevertheless, proximal thickening and distal atrophy, as well as clinical signs, did not sufficiently improve. A very likely explanation is that clotrimazole is a broad-spectrum, topical, nonsystemic, antifungal drug. Only very small amounts are absorbed by the mucosa and thus reach the affected nerve [24]. In this case, local thickening of the pharyngeal branch represents inflammatory and degenerative changes within the nerve caused by invasion of fungal hyphae. This leads to a chronic interstitial neuritis and a compression of nerve fibers, which explains the peripheral degenerative atrophy [21]. Unfortunately, it was not possible to get histological sections from this pony to prove this hypothesis.

In future, it would be interesting to use Canesten^®^ Gyn Once^d^, which has a three-day depot effect, for the treatment of guttural pouch mycosis. Thus, retreatment and sedation would have to be performed only every third day. Manipulation could be reduced and this would be beneficial for both the horse and the veterinarian.

Depending on the severity of the lesion and clinical symptoms, guttural pouch mycosis in combination with neurological deficits has a guarded to hopeless prognosis. However, spontaneous recovery has been reported [9]. Dysphagia especially is very hard to manage and requires a lot of patience, care, treatment and support to meet the nutritional requirements of the horse [10]. Enteral nutrition via nasogastric tube would have been necessary, very expensive, time-consuming, and risky. The average recovery time of horses with neurologic deficits due to guttural pouch mycosis lasts from 3 to 18 months [10,14,22,23]. Even if all the fungal hyphae can be eliminated, the slow healing process of the nerves will be the major issue. Unfortunately, the mare was not able to maintain her body condition, vital signs and pulmonary function and thus had to be euthanized.

## Conclusion

The clinical symptom of dysphagia always requires a close investigation of the upper respiratory tract and both guttural pouches. Even small, focal, one-sided lesions on the pharyngeal branch of the vagus nerve, as it crosses the guttural pouch, lead to severe regurgitation and dysphagia. Management is difficult and a standard approach to treatment of guttural pouch mycosis does not exist. Local irrigation with a 0.08% clotrimazole emulsion is able to clear the fungal infection at mucosal site, but it does not improve neuronal function. The use of a three-day depot clotrimazole emulsion requires further evaluation.

### Endnotes

^a^Detomidine: Cepesedan^®^, CP-Pharma, Burgdorf, Germany.

^b^Amoxicillin: Amoxisel^®^, Selectavet, Fischer GmbH, Munich, Germany.

^c^Metapyrin: Metamizol^®^, Serumwerke AG, Bernburg, Germany.

^d^Clotrimazole: Canesten^®^, Gyn, Bayer Vital GmbH, Leverkusen, Germany.

^e^Flunixine meglumine: Finadyne^®^, MSD Tiergesundheit, Unterschleißheim, Germany.

## Competing interests

The authors declare that they have no competing interests.

## Authors’ contributions

AE drafted the manuscript, carried out the clinical work-up and helped to perform the endoscopic examination. AS helped to draft the manuscript and supervised the management of the patient. SR helped to draft the manuscript and carried out the endoscopic examination. AU supervised the diagnostics and daily treatment of the pony. VW carried out the histopathological examination of the biopsy. GFS supervised the clinical work, helped with the clinical work-up and reviewed the manuscript. All authors read and approved the final manuscript.
